# REFRAMING STUDENT EXPERIENCES AND ATTITUDES TOWARDS WORKING WITH OLDER ADULTS

**DOI:** 10.1093/geroni/igac059.2734

**Published:** 2022-12-20

**Authors:** Iveris Martinez

**Affiliations:** California State University, Long Beach, Long Beach, California, United States

## Abstract

We are experiencing a shortage of trained health and social service providers to meet the needs of an aging society. However, few students have positive opportunities to work with older adults in their training. If they interact with older adults it is usually in end-of-life and nursing home care settings. We therefore need to find creative ways to motivate students in these fields to choose to work with older adults. We recruited students from health and social services programs to implement four health promotion projects at an older adult low-income residential community. We asked students (Nf22) to reflect on their experiences, and analyzed responses using a grounded theory approach. Myths regarding working with older adults included that they were mean, difficult, not technologically savvy, nor physically active. Initially nervous and uncertain about working with older adult prior to their experience, students gained confidence and had fun. They reported rewarding experiences, built relationships, and learned the benefits of prevention programs for older adults, and reconsidering their career trajectories to focus on working with older adults. Encouraging positive student experiences working with older adults can help prepare to develop the health and human services workforce for an aging society.

